# Chlorinated Azaphilone Pigments with Antimicrobial and Cytotoxic Activities Isolated from the Deep Sea Derived Fungus *Chaetomium* sp. NA-S01-R1

**DOI:** 10.3390/md16020061

**Published:** 2018-02-13

**Authors:** Weiyi Wang, Yanyan Liao, Ruixuan Chen, Yanping Hou, Wenqian Ke, Beibei Zhang, Maolin Gao, Zongze Shao, Jianming Chen, Fang Li

**Affiliations:** 1 State Key Laboratory Breeding Base of Marine Genetic Resources, Key Laboratory of Marine Genetic Resources, Fujian Key Laboratory of Marine Genetic Resources, Fujian Collaborative Innovation Centre for Exploitation and Utilization of Marine Biological Resources, Third Institute of Oceanography, State Oceanic Administration, Xiamen 361005, China; 18606928642@163.com (R.C.); houyanping@tio.org.cn (Y.H.); 15260587285@163.com (W.K.); zbb953372968@163.com (B.Z.); g17785208284@163.com (M.G.); shaozongze@tio.org.cn (Z.S.); 2 Key Laboratory of Urban Environment and Health, Institute of Urban Environment, Chinese Academy of Sciences, Xiamen 361021, China; yyliao@iue.ac.cn; 3 Institute of Oceanography, Minjiang University, Fuzhou 350108, China

**Keywords:** *Chaetomium*, chaephilone, chaetoviridide, antimicrobial, cytotoxic

## Abstract

Four novel compounds, chaephilone C (**1**), chaetoviridides A–C (**2**–**4**), were obtained from the culture of a deep sea derived fungus *Chaetomium* sp. NA-S01-R1, together with four known compounds—chaetoviridin A (**5**), chaetoviridine E (**6**), chaetomugilin D (**7**) and cochliodone A (**8**). Their structures, including absolute configurations, were assigned based on NMR, MS and time-dependent density functional theory (TD-DFT) ECD calculations. A plausible biogenetic pathway for compounds **1**–**3** was proposed. Compounds **2** and **3** exhibited antibacterial activities against *Vibrio rotiferianus* and *Vibrio vulnificus*. Compounds **1**, **3** and **4** displayed similar anti-methicillin resistant *Staphylococcus aureus* (anti-MRSA) activities in comparison to chloramphenicol. Compound **2** showed the most potent cytotoxic activities towards the Hep G2 cell and compounds **1** and **3** demonstrated relatively stronger cytotoxic activities than the other compounds against the HeLa cell.

## 1. Introduction

Fungi are well-known as prolific producers of secondary metabolites with structural and biological diversity. Azaphilones are a family of fungal pigments characterized by a highly oxygenated pyrano-quinone bicyclic core. The colored azaphilone derivatives are produced by species of ascomyceteous and basidiomyceteous fungi, including the genera *Penicillium*, *Aspergillus*, *Chaetomium*, *Talaomyces*, *Emericella*, *Epicoccum*, *Pestalotiopsis*, *Phomopsis*, *Monascus* and *Hypoxylon*. Several azaphilones are unique to one species and regarded as chemotaxonomical indicators. Azaphilones have been reported to exhibit a wide range of biological activities, such as antimicrobial, cytotoxic, antiviral and anti-inflammatory activities, as well as inhibitors of heat shock proteins (Hsp 90), MDM2-p53 interaction and gp120-CD4 binding [1].

**Figure 1:**
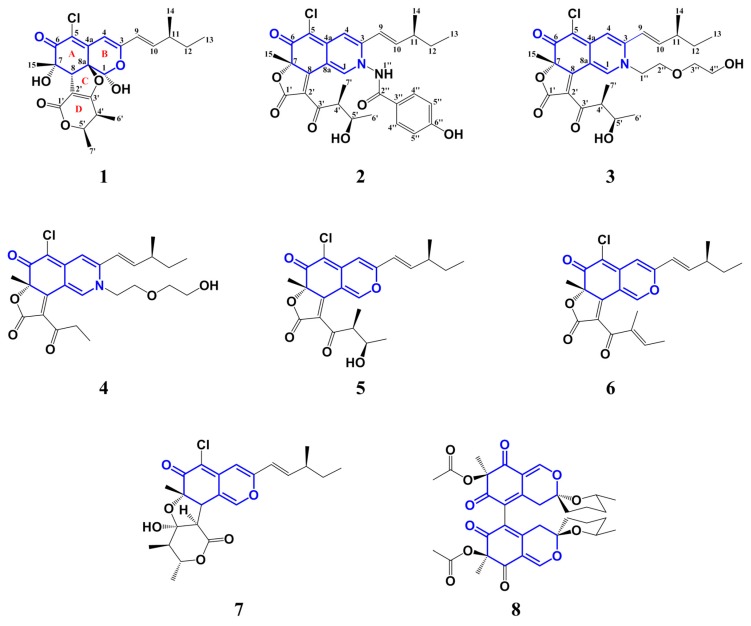
Structure of compounds **1**–**8**.

As part of our ongoing efforts to discover bioactive compounds from marine-derived microorganisms, a *Chaetomium* sp. strain NA-S01-R1, isolated from the seawater sample at a depth of 4050 m (20°25′11.0321′′ N, 155°51′22.1549′′ E) in the West Pacific Ocean in 2017, attracted our attention. Studies on bioactive constituents of its pigment fraction led to the isolation of four novel chlorinated compounds, chaephilone C (**1**) and chaetoviridides A–C (**2**–**4**), together with four known compounds, chaetoviridin A (**5**), chaetoviridine E (**6**), chaetomugilin D (**7**) and cochliodone A (**8**) (Figure 1). Compounds **5**–**7** were azaphilones bearing a five-membered lactone and a fused tetrahydrofuran/*δ*-lactone respectively and compound **8** was a dimeric bis-spiro-azaphilone derivative. Their structures were elucidated through spectroscopic methods and cytotoxic and antimicrobial activities were both evaluated.

## 2. Results and Discussion

Chaephilone C (**1**), chaetoviridides A–C (**2**–**4**) were characterized as azaphilone derivatives with a chlorine atom at C-5, a methyl unit at C-7 and a branched pentenyl side chain at C-3. Chromium trioxide oxidation of compounds **1**–**5** gave 2-methylbutanoic acid showing (+)-rotation. Thus, the stereochemistry of C-11 for compounds **1**–**4** in the pentenyl side chain was established as (*S*).

**Figure 2:**
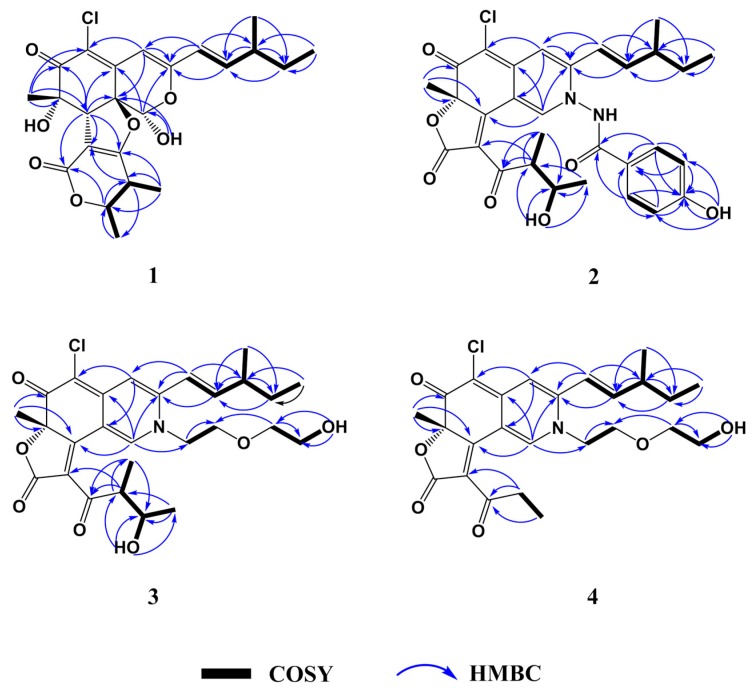
Key COSY and HMBC correlations of compounds **1**–**4**.

Chaephilone C (**1**) was obtained as a yellow amorphous solid. Its molecular formula was established as C_23_H_27_O_7_Cl by high-resolution electrospray ionization mass spectroscopy (HR-ESI-MS) (*m*/*z* 449.1373 [M − H]^−^; calcd. for C_23_H_26_O_7_Cl, 449.1367; ∆ + 1.3 ppm) and the ratio of isotope peaks ([M − H]^−^/[M − H + 2]^−^), implying ten degrees of unsaturation. The ^13^C/DEPT and HSQC spectrum revealed the presence of one primary methyl group (C-13), three secondary methyl groups (C-6′, C-7′ and C-14), one tertiary methyl group (C-15), one methylene group (C-12), five sp^3^-hybridized methine groups (C-1, C-4′, C-5′, C-8 and C-11) including two oxygen-bearing carbons (C-1 and C-5′), three sp^2^-hybridized methine groups (C-4, C-9 and C-10), two sp^3^-hybridized quaternary oxygen-bearing carbons (C-7 and C-8a), five sp^2^-hybridized quaternary carbons (C-3, C-4a, C-5, C-2′ and C-3′) including one oxygen-bearing carbon (C-3′) and two carbonyl carbons (C-6 and C-1′) (Figures S3 and S6). The ^1^H-^1^H COSY spectrum allowed the elucidation of two partial units as shown by bold-faced lines in Figure 2. The geometrical configuration of the double bond (C-9–C-10) was deduced as *trans* from the coupling constant of the olefinic protons ^3^*J*_9,10_ (15.95) (Table 1). The connection of these units and the remaining groups was established based on the HMBC correlations as shown in Figure 2. The 3-methyl-1-pentenyl chain was connected to C-3 by HMBC correlations of H-9 with C-3 and C-4 and of H-10 with C-3. The connection of a chlorine atom to C-5 was reasonable from its chemical shift (*δ*_C_ 124.2). Therefore, the planar structure of **1** was assigned and named chaephilone C (**1**).

**Figure 3:**
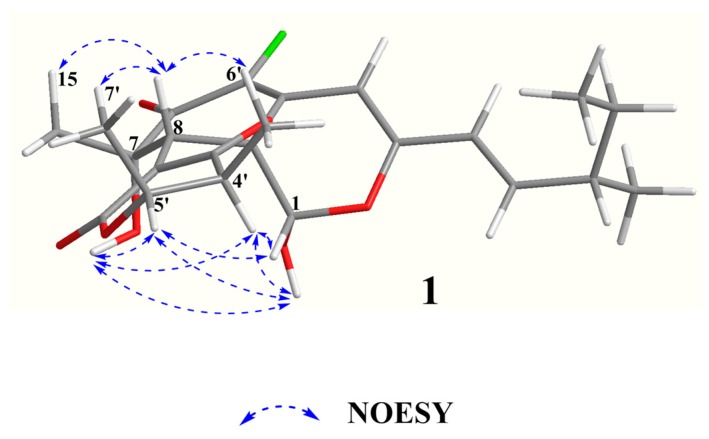
Key NOESY correlations of compound **1**.

**Figure 4:**
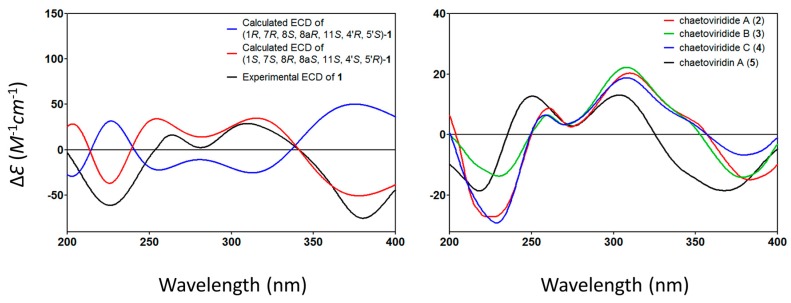
Calculated ECD of compound **1** and Experimental ECD of compounds **1**–**5**.

The relative configuration of **1** was elucidated based on NOESY spectra (Figure 3). The strong NOESY correlations of OH-1 with OH-7, H-4′ and H-5′, of H-1 with H-4′ and H-5′, of H-8 with H-15, H-6′ and H-7′ and no NOE correlations of H-8 with H-4′ and H-5′, indicated that the unit of C-1-OH, OH-7, H-4′ and H-5′ faced to the same side of the fused A/C/D rings and H-8, H-15, H-6′ and H-7′ oriented to the opposite side. Therefore, two possible isomers of (1*S*, 7*S*, 8*R*, 8a*S*, 11*S*, 4′*S*, 5′*R*)-**1** and (1*R*, 7*R*, 8*S*, 8a*R*, 11*S*, 4′*R*, 5′*S*)-**1** were proposed and their ECD spectra were calculated by time-dependent density functional theory (TD-DFT). The experimental ECD spectrum of **1** was in good agreement with the calculated ECD spectrum of (1*S*, 7*S*, 8*R*, 8a*S*, 11*S*, 4′*S*, 5′*R*)-**1** (Figure 4). Thus, the absolute configuration at C-1, C-7, C-8, C-8a, C-4′ and C-5′ of **1** was established as *S*, *S*, *R*, *S*, *S*, *S* and *R*, respectively and the structure of **1** was shown in Figure 1.

Chaetoviridide A (**2**) was obtained as a red amorphous solid. Its molecular formula was determined as C_30_H_31_N_2_O_7_Cl by HR-ESI-MS (*m*/*z* 565.1735 [M − H]^−^; calcd. for C_30_H_30_N_2_O_7_Cl, 565.1742; ∆ − 1.2 ppm) and the ratio of isotope peaks ([M − H]^−^/[M − H + 2]^−^), implying sixteen degrees of unsaturation. Analysis of ^1^H-^1^H COSY spectrum allowed the assignment of the 3-methyl-1-pentenyl and 2-butanol-3-yl moieties. 3-methyl-1-pentenyl was attached to C-3 by HMBC correlations to C-3 from H-9 and H-10. 2-butanol-3-yl was connected to the conjugated carbonyl C-3′ by HMBC correlations of H-4′ to C-2′ and of H-7′ to C-3′ (Figure 2). Partial spectra data of **2** were similar to chaetoviridin A, except that C-1, C-3 and C-8a were shifted to upfield and C-4a to downfield (Table 1). Chemical shift differences and the detailed 2D NMR (^1^H-^1^H COSY, HSQC and HMBC) correlations of the unassigned carbons suggested the nitrogen at position of **2** bearing a *p*-hydroxybenzamide moiety which was confirmed by the NOE correlations from H-1′′ to H-1, H-9, H-10 and H-4′′ (Figure S17). Therefore, the planar structure of **2** was deduced and named chaetoviridide A (**2**).

Chaetoviridide B (**3**) was obtained as a red amorphous solid. Its molecular formula was determined as C_27_H_34_NO_7_Cl by HR-ESI-MS (*m*/*z* 520.2106 [M + H]^+^; calcd. for C_27_H_35_NO_7_Cl, 520.2102; ∆ + 0.8 ppm) and the ratio of isotope peaks ([M + H]^+^/[M + H + 2]^+^), implying eleven degrees of unsaturation. The ^1^H-^1^H COSY spectrum allowed the elucidation of four partial units as shown by bold-faced lines in Figure 2. The connection of these units and the remaining groups was established based on the HMBC correlations as shown in Figure 2. By comparison of NMR data with chaetoviridin A, **3** was characterized as a nitrogenated chaetoviridin A derivative with a 2-hydroxyethoxy-ethyl group attached to N-2, which was confirmed by HMBC correlations from H-1 to C-1′′. The planar structure of **3** was established as N-2-(hydroxyethoxy)ethyl chaetoviridin A and named chaetoviridide B (**3**).

Chaetoviridide C (**4**) was obtained as a red amorphous solid. Its molecular formula was determined as C_25_H_30_NO_6_Cl by HRESI-MS (*m*/*z* 476.1828 [M + H]^+^; calcd. for C_25_H_31_NO_6_Cl, 476.1840; ∆ − 2.5 ppm) and the ratio of isotope peaks ([M + H]^+^/[M + H + 2]^+^), indicating eleven degrees of unsaturation attributed to 3 rings and 8 double bonds. By comparison of 1D NMR data with chaetoviridide B (**3**) and analysis of 2D NMR data (Figures S25–S30), **4** were characterized as a chaetoviridide B analog with an ethyl group attached to C-3′ instead of the 2-butanol-3-yl moiety. Therefore, the planar structure of **4** was deduced and named chaetoviridide C (**4**).

The optical rotation values of **2**–**4** exhibited the same sign compared to that of chaetoviridin A (**5**) isolated here, ${\left[\alpha \right]}_{D}^{20.0}\;85\;\left(c\;0.15,\;\mathrm{MeOH}\right)$. In addition, in CD spectra, the negative cotton effects at wavelengths of 225 and 378 nm and positive of 256 and 305 nm for **2**–**4**, which were also similar to those of chaetoviridin A (**5**). Furthermore, the NMR data of C-7, C-4′ and C-5′ for **2**–**3** and of C-7 for **4** were almost identical to those of chaetoviridin A (**5**). The above evidence sufficiently allowed the assignment of the remaining asymmetric centers (7*S*, 4′*S*, 5′*R*). Therefore, the absolute configuration of **2**–**4** was established and shown in Figure 1.

**Scheme 1:**
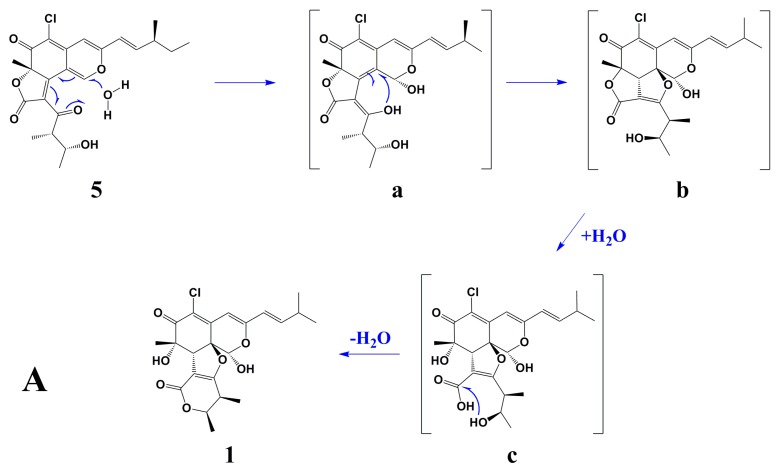

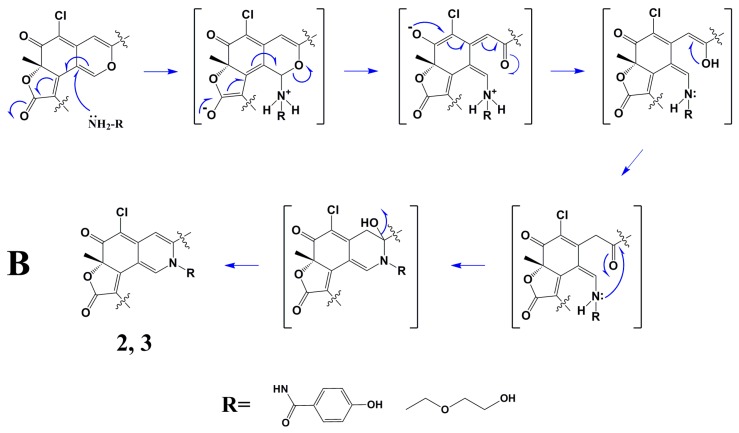
Postulated Biogenetic Pathway for **1**–**3**. (**A**) shows the postulated biogenetic pathway for **1**; (**B**) shows the plausible mechanism for **2** and **3** via a Schiff base formation and dehydration reaction.

Scheme 1A showed the postulated biogenetic pathway for **1**, which started with hydration of chaetoviridin A (**5**) to form intermediate **a**, followed by attack of 3′-OH on C-8a, hydrolytic opening of the γ-lactone [2] and post dehydration to obtain chaephilone C (**1**). Scheme 1B showed the plausible mechanism for **2** and **3** via a Schiff base formation and dehydration reaction which has been reported previously [3,4,5].

The known compounds (**5**–**8**) were identified as chaetoviridin A (**5**) [6], chaetoviridine E (**6**) [7], chaetomugilin D (**7**) [8] and cochliodone A (**8**) [7] respectively, by comparing their spectroscopic data with those reported in the literature.

The antimicrobial and cytotoxic activity of compounds **1**–**8** was evaluated using cell lines of A549, HeLa and Hep G2 and strains of *Vibrio vulnificus* MCCC E1758, *Vibrio rotiferianus* MCCC E385 and *Vibrio campbellii* MCCC E333, methicillin-resistant *Staphylococcus aureus* (MRSA) (ATCC 43300, CGMCC 1.12409) (Table 2). For strains of *Vibrio*, compounds **1**–**8** showed weaker activities than the positive control erythromycin. Compounds **2** and **3** exhibited relatively stronger activities than the other compounds against *V. rotiferianus* and *V. vulnificus* with MIC values ranging from 7 to 8 μg/mL, respectively. For strains of MRSA, **1**, **3** and **4** showed similar activities in comparison to the positive control chloramphenicol with MIC values ranging from 7 to 8 μg/mL. For cytotoxicity, compounds **1**–**8** displayed weaker activities than the positive control doxorubicin. Compound **2** exhibited the most potent cytotoxic activities towards Hep G2 cell with IC_50_ value as 3.9 μM and compounds **1** and **3** demonstrated relatively stronger activities against HeLa cells with IC_50_ values ranging from 5–8 μM.

## 3. Materials and Methods

### 3.1. General Experimental Procedures

1D NMR and 2D NMR spectra were measured on a Bruker Avance 600 MHz spectrometer. HRESIMS was carried out on a Xevo G2 Q-TOF mass spectrometer with an electrospray ionization source (Waters, Milford, MA, USA). Optical rotations were measured with a P-1020 digital polarimeter (JASCO Corporation, Tokyo, Japan). CD spectra were measured on a J-715 spectropolarimeter (JASCO Corporation). Optical rotations were recorded on a Rudolph IV Autopol automatic polarimeter (Hackettstown, NJ, USA). The UV spectra were recorded on a UV-1800 spectrophotometer (Shimadzu, Kyoto, Japan). The high-performance liquid chromatography (HPLC) analysis was performed on a 1200 system (Agilent, Santa Clara, CA, USA). Thin-layer chromatography (TLC) plates (5 × 10 cm) were performed on GF_254_ (Qingdao Marine Chemical Co. Ltd., Qingdao, China) plates. For column chromatography (CC), RP-C18 (ODS-A, 50 µm, YMC, Kyoto, Japan), silica gel (200–300 mesh, 300–400 mesh, Qingdao Marine Chemical Co. Ltd., Qingdao, China) and Sephadex LH-20 (GE Healthcare Bio-Science AB, Pittsburgh, PA, USA) were used. Semi-preparative HPLC was run with a P3000 pump (CXTH, Beijing, China) and a UV3000 ultraviolet-visible detector (CXTH, Beijing, China), using a preparative RP-C18 column (5 µm, 20 × 250 mm, YMC, Kyoto, Japan).

### 3.2. Fungal Material

Strain NA-S01-R1 of *C*. sp. was identified by ITS sequence homology (99% similarity with *Chaetomium globosum* strain GYY2(1) with Genbank Accession No. KM268652.1 (max score 974, *e* value 0.0, query cover 97%)). The fungal strain was inoculated into a 15 mL centrifuge tube containing 5 mL of potato dextrose (PD) medium and cultured at 28 °C at 120 rpm for 3 days. Total genomic DNA was extracted as described by Lai et al. [9]. The internal transcribed spacer (ITS) region of rDNA was amplified by PCR using primers ITS1 (5′-TCCGTAGGTGAACCTGCGG-3′) and ITS4 (5′-TCCTCCGCTTATTGATATGC-3′). The PCR mixture consisted of 12.5 μL Taq premix (TaKaRa, Beijing, China), 0.25 μL (10 μM) of each primer, 0.75 μL dimethyl sulfoxide (DMSO), 10.25 μL dd. H_2_O and 1 μL DNA template. After denaturation at 95 °C for 4 min, amplification was performed with 32 cycles of 30 s at 95 °C, 30 s at 55 °C and 40 s at 72 °C and a final extension at 72 °C for 7 min. The ITS1-5.8S-ITS2 rDNA sequence of the fungus has been submitted to GenBank with the accession number MG786198. A voucher specimen was deposited at the Third Institute of Oceanography, SOA, China. The working strain was prepared on potato dextrose agar (PDA) slants and stored at 4 °C.

### 3.3. Fermentation, Extraction and Isolation

Strain NA-S01-R1 was cultured on PDA plates at 28 °C for 3 days. Then, six plugs (5 mm diameter) were transferred to 12 Erlenmeyer flasks (1 L), each containing 500 mL Czapek’s medium (NaNO_3_ 3.0 g/L, KH_2_PO4 1.0 g/L, MgSO_4_·7H_2_O 0.5 g/L, sucrose 30 g/L, FeSO_4_ 0.01 g/L and KCl 0.5 g/L) in sterile conditions. Erlenmeyer flasks were shaken on a rotary shaker at 28 °C and 120 rpm for 3 days to form seed cultures (1 × 10^8^ spores/mL). Next, seed cultures (45 × 100 mL) were transferred to flasks (45 × 1 L) containing 105 g of rice and 45 g of millet per flask. After 25 days, the fermented broth was dried, smashed and extracted with ethyl acetate (EtOAc). The organic solvent was evaporated under reduced pressure to afford the EtOAc extract (150 g), which was partitioned by 90% methanol in water and then extracted with petroleum ether (PE) three times. The methanol layer was concentrated to provide a defatted extract (50 g), which was subject to Sephadex LH-20 column chromatography, eluting with methanol to yield eight fractions, A–H, of which fraction B (500 mg) was in red color and exhibited better antibacterial and cytotoxic than the other fractions. Therefore, fraction B was applied to silica gel column chromatography using PE-acetone (9:1, 8.5:1.5, 8:2, 7.5:2.5, 6.5:3.5, *V*:*V*) to yield five subfractions, B1–B5. Fraction B1 was further purified by semi-preparative HPLC (90% methanol in H_2_O, flow rate 8 mL/min) and Sephadex LH-20 (90% methanol in water) to give compounds **7** (5 mg) and **4** (3.2 mg). Fraction B2 was further separated by semi-preparative HPLC (85% acetonitrile in H_2_O, flow rate 8 mL/min) to obtain compounds **1** (5 mg) and **8** (3 mg). Fraction B3 was subjected to Sephadex LH-20 (85% methanol in water) to yield compounds **3** (4.8 mg) and **5** (50 mg). Fraction B4 was purified by preparative HPLC (80% methanol in water) and Sephadex LH-20 to obtain compounds **2** (3.5 mg) and **6** (10 mg).

Chaephilone C (**1**): yellow amorphous solid; ${\left[\alpha \right]}_{D}^{20.0}$ −125 (*c* 0.04, MeOH); UV λ_max_ (methanol) nm (log *ε*): 300 (4.37), 380 (4.32); ^1^H NMR and ^13^C NMR data are shown in Table 1; HR-ESI-MS: *m*/*z* 449.1373 [M − H]^−^ (Calcd. for 449.1367, C_23_H_26_O_7_Cl, ∆ + 1.3 ppm).

Chaetoviridide A (**2**): red amorphous solid; ${\left[\alpha \right]}_{D}^{20.0}$ +105 (*c* 0.03, MeOH); UV λ_max_ (methanol) nm (log *ε*): 297 (4.56), 380 (4.43); ^1^H NMR and ^13^C NMR data are shown in Table 1; HR-ESI-MS: *m*/*z* 565.1735 [M − H]^−^ (Calcd. for 565.1742, C_30_H_30_N_2_O_7_Cl, ∆ − 1.2 ppm).

Chaetoviridide B (**3**): red amorphous solid; ${\left[\alpha \right]}_{D}^{20.0}$ +95 (*c* 0.03, MeOH); UV λ_max_ (methanol) nm (log *ε*): 267 (4.55), 299 (4.58), 384 (4.53); ^1^H NMR and ^13^C NMR data are shown in Table 1; HR-ESI-MS: *m*/*z* 520.2106 [M + H]^+^ (Calcd. for 520.2102, C_27_H_35_NO_7_Cl, ∆ + 0.8 ppm).

Chaetoviridide C (**4**): red amorphous solid; ${\left[\alpha \right]}_{D}^{20.0}$ +90 (*c* 0.03, MeOH); UV λ_max_ (methanol) nm (log *ε*): 299 (4.42), 380 (4.35); ^1^H NMR and ^13^C NMR data are shown in Table 1; HR-ESI-MS: *m*/*z* 476.1828 [M + H]^+^ (Calcd. for 476.1840, C_25_H_31_NO_6_Cl, ∆ − 2.5 ppm).

### 3.4. ECD Calculation

Conformational analysis was initially performed using Confab [10] with systematic search at MMFF94 force field for undetermined relative configurations of compound **1**. Room-temperature equilibrium populations were calculated according to the Boltzmann distribution law Equation (1). Dominative conformers with Boltzmann-based population over 1% were delivered to subsequent Quantum Mechanics (QM) calculations. The energies and populations of conformers were provided in Table S1.

(1) $$\frac{N_{i}}{N}=\frac{g_{i}e^{-\frac{E_{i}}{k_{B}T}}}{\sum g_{i}e^{-\frac{E_{i}}{k_{B}T}}}$$

*N_i_* is the number of conformer *i* with energy *E_i_* and degeneracy *g_i_* at temperature *T* and *k*_B_ is the Boltzmann constant.

The theoretical calculations were carried out using Gaussian 09. At first, conformers were optimized at PM6 using semi-empirical theory method. The conformers with Boltzmann-based population lower than 1% were again filtered out and the remaining chosen for further optimization at B3LYP/6-311G(d,p) in methanol using the integral equation formalism polarizable continuum model (IEFPCM) (Table S2). Vibrational frequency analysis confirmed the stable structures. Under the same condition, the ECD calculation was conducted using TD-DFT. Rotatory strengths for a total of 30 excited states were calculated. The ECD spectrum was simulated in SpecDis [11] by overlapping Gaussian functions for each transition.

### 3.5. Antibacterial Assay

Antibacterial activities against *V. rotiferianus* (MCCC E385), *V. vulnificus* (MCCC E1758), *V. campbellii* (MCCC E333) and MRSA (ATCC 43300, CGMCC 1.12409), were tested by continuous dilution in 96-well plates using resazurin as a surrogate indicator. Blue resazurin was reduced by metabolically active bacteria to pink resorufin. A mid-logarithmic-phase tested strain was added at a starting inoculum of 5 × 10^5^ CFU/mL to the plate containing tested compound (final concentration ranging from 125 to 0.98 μg/mL in two-fold dilution) plus 10% resazurin solution (6.75 mg/mL in sterile water). The foil covered plate was incubated for 24 h with shaking at 37 °C. The MIC value was determined to be the lowest concentration that did not induce the color change [12,13,14] by observing the blue-to-pink color change.

### 3.6. Cytotoxicity Assay

A549 (adenocarcinomic human alveolar basal epithelial cell), Hela (cervical cancer cell) and Hep G2 (human liver cancer cell) cells were maintained in F-12K, DMEM and MEM medium respectively and supplied with 10% FBS, 100 U/mL of penicillin and 100 mg/mL of streptomycin [15]. Cells were grown in a humidified chamber with 5% CO_2_ at 37 °C. For cytotoxicity assays, cells were seeded at a density of 5000 cells per well in 96-well plates, grown at 37 °C for 12 h and then treated with tested compound at five different concentrations (100 μL medium/well). The cytotoxicity was measured by Cell Counting Kit-8 (CCK-8) (DOJINDO) at 48 h post-treatment, following the manufacturer’s instructions.

CCK-8 assay is based on the conversion of a tetrazolium salt, 2-(2-methoxy-4-nitrophenyl)-3-(4-nitrophenyl)-5-(2,4-disulfophenyl)-2*H*-tetrazolium, monosodium salt (WST-8) and a water-soluble formazan dye, upon reduction by dehydrogenases in the presence of an electron mediator [16]. WST-8 is reduced by dehydrogenases in cells to give an orange colored product (formazan). The amount of the formazan dye is directly proportional to the number of living cells.

In brief, 10 μL of CCK-8 solution was added to each well of the 96-well plates. After incubation at 37 °C for 2 h, the absorbance at 450 nm was measured using a SpectraMAX M5 microplate reader. Wells with only culture medium and CCK-8 solution were used to determine the background and cells treated with DMSO were included as the negative controls [15].

## 4. Conclusions

In current research, we have isolated four chlorinated novel compounds, chaephilone C (**1**), chaetoviridide A–C (**2**–**4**), together with four known azaphilone derivatives, chaetoviridin A (**5**), chaetoviridine E (**6**), chaetomugilin D (**7**) and cochliodone A (**8**). The absolute configuration of **1**–**4** was elucidated by TD-DFT ECD method and plausible biogenetic pathway for **1**–**3** was proposed. Remarkably, compound **2** and **3** showed antibacterial activities against VR and VV respectively. Compounds **1**, **3** and **4** displayed similar anti-MRSA activities in comparison to chloramphenicol. Compounds **1** and **3** demonstrated relatively stronger cytotoxic activities than the other compounds against HeLa cell and compound **2** showed the most potent cytotoxic activities towards Hep G2 cell with IC_50_ below 5 μM. Further studies should be conducted to elucidate the antibacterial and cytotoxic mechanism of the related compounds as well as their roles in the life cycle of the deep sea ecological system.

## Figures and Tables

**Table 1:** ^1^H NMR data (600 MHz) and ^13^C NMR data (150 MHz) for compounds **1**–**4** (DMSO-*d*_6_).

Position	1		2		3		4	
	δ_H_, Mult. (*J* in Hz)	δ_C_, Type	δ_H_, Mult. (*J* in Hz)	δ_C_, Type	δ_H_, Mult. (*J* in Hz)	δ_C_, Type	δ_H_, Mult. (*J* in Hz)	δ_C_, Type
1	5.38, d (5.19)	93, CH	8.51, s	142.9, CH	8.59, s	142.4, CH	8.68, s	143.2, CH
3		155.6, C		149.3, C		149.1, C		145.5, C
4	6.01, s	99.5, CH	6.9, s	107.8, CH	6.79, s	109.5, CH	6.79, s	109.6, CH
4a		137.7, C		144.6, C		145.4, C		149.1, C
5		124.2, C		110.9, C		111.1, C		110.8, C
6		190, C		181, C		180.1, C		180.1, C
7		72.9, C		88.4, C		88.7, C		88.7, C
8	3.29, m	48.6, CH		165.4, C		166.8, C		168.3, C
8a		85.7, C		99.8, C		97.5, C		97.5, C
9	6.18, d (15.95)	123.7, CH	6.23, d (15.81)	118.1, CH	6.63, d (15.41)	120.8, CH	6.63, d (15.41)	120.9, CH
10	6.33, m	144.2, CH	6.61, dd (15.81, 8.03)	149.2, CH	6.47, d (15.41, 7.89)	148.9, CH	6.47, dd (15.41, 7.89)	148.8, CH
11	2.23, m	38.2, CH	2.18, m	39, CH	2.33, m	38.9, CH	2.33, m	38.9, CH
12	1.36, m	29.1, CH_2_	1.3, m	28.7, CH_2_	1.44, m	29, CH_2_	1.44, m	29, CH_2_
13	0.86, t (7.44)	12.1, CH_3_	0.69, t (7.28)	11.8, CH_3_	0.88, t (7.44)	12.1, CH_3_	0.89, t (7.43)	12.1, CH_3_
14	1.02, d (6.74)	19.5, CH_3_	0.95, d (6.6)	19.6, CH_3_	1.07, d (6.79)	19.6, CH_3_	1.08, d (6.79)	19.6, CH_3_
15	1.31, s	24, CH_3_	1.6, s	26.7, CH_3_	1.56, s	26.8, CH_3_	1.57, s	26.9, CH_3_
1′		164.5, C		168.9, C		169.1, C		169.1, C
2′		100.6, C		124.4, C		124.1, C		122, C
3′		175.1, C		200.9, C		200.9, C		197.1, C
4′	2.78, m	35.2, CH	3.53, m	50.7, CH	3.5, m	50.9, CH	3.06, dq (19.05, 7.10)	35.2, CH_2_
4′							2.84, dq (19.05, 7.10)	35.2, CH_2_
5′	4.29, m	78.6, CH	3.56, m	69.5, CH	3.62, m	69.5, CH	0.99, t (7.12)	7.8, CH_3_
6′	0.98, d (7.19)	12.3, CH_3_	0.93, d (6.6)	21.5, CH_3_	0.95, d (6.24)	21.5, CH_3_		
7′	1.36, d (6.39)	18.5, CH_3_	0.94, d (6.6)	12.7, CH_3_	0.99, d (6.79)	12.9, CH_3_		
1′′			12.32, s		4.2, m	54.2, CH_2_	4.33, m	54.1, CH_2_
1′′					4.35, m	54.2, CH_2_	4.23, m	54.1, CH_2_
2′′				165.7, C	3.72, m	68.8, CH_2_	3.76, m	68.8, CH_2_
2′′					3.78, m	68.8, CH_2_		
3′′				121.2, C	3.45, m	72.9, CH_2_	3.45, m	72.9, CH_2_
4′′			7.83, d (8.53)	130.5, CH	3.46, br t (3.03)	60.6, CH_2_	3.46, m	60.6, CH_2_
5′′			6.92, d (8.53)	116, CH				
6′′				162.4, C				
OH-1	8.19, d (5.19)							
OH-7	5.72, s							
OH-5′			4.64, d (5.27)		4.65, br d (5.32)			
OH-4′′					4.57, br s		4.54, br s	
OH-6′′			10.43, s					

**Table 2:** Antimicrobial and cytotoxic activities of compounds **1**–**8**. Data are expressed as mean ± SD values of three independent experiments, each made in triplicate.

Compound	MIC (μg/mL)					IC_50_ (μM)		
	VV	VR	VC	MRSA 1	MRSA 2	A549	HeLa	Hep G2
1	32.2 ± 1.5	NA	30.1 ± 1.2	**7.8 ± 0.8**	**7.6 ± 0.5**	15.7 ± 1.1	**7.7 ± 0.9**	20.2 ± 1.6
2	30.5± 1.2	**7.3 ± 0.8**	32.7± 1.9	15.5 ± 0.8	15.7 ± 0.9	15.2 ± 0.9	12.3 ± 0.6	**3.9 ± 0.7**
3	**7.4 ± 0.3**	31.3 ± 0.2	32.3 ± 0.3	**7.3 ± 0.6**	**7.8 ± 0.8**	16.3 ± 0.7	**5.6 ± 0.9**	18.2 ± 1.0
4	15.7 ± 0.6	15.3 ± 0.7	NA	**7.7 ± 0.8**	**7.6 ± 0.4**	23.1 ± 0.9	17.7 ± 0.3	22.2 ± 0.6
5	16.6 ± 0.4	32.3 ± 0.3	16.0 ± 0.4	15.2 ± 0.4	16.1 ± 0.3	40.0 ± 0.3	42.2 ± 0.5	38.1 ± 1.1
6	15.4 ± 0.8	NA	NA	NA	NA	37.7 ± 0.3	NA	41.2 ± 0.6
7	32.4 ± 0.5	15.3 ± 0.2	NA	32.2 ± 0.3	32.4 ± 0.1	NA	NA	NA
8	15.3 ± 0.5	32.7 ± 0.2	NA	15.9 ± 0.3	16.3 ± 0.5	28.1 ± 1.0	20.7 ± 0.4	23.2 ± 0.7
erythromycin	2.0 ± 0.0	3.9 ± 0.1	7.7 ± 0.2	NT	NT	NT	NT	NT
chloramphenicol	NT	NT	NT	7.6 ± 0.2	7.5 ± 0.1	NT	NT	NT
doxorubicin	NT	NT	NT	NT	NT	0.1 ± 0.0	0.5 ± 0.1	1.1 ± 0.1

VV: *V. vulnificus* MCCC E1758; VR: *V. rotiferianus* MCCC E385; VC: *V. campbellii* MCCC E333; MRSA 1: methicillin-resistant *S. aureus* ATCC 43300; MRSA 2: methicillin-resistant *S. aureus* CGMCC 1.12409; NA: no activity at the concentration of 50 μg/mL (antibacterial) or 50 μM (cytotoxic); NT: not tested. Figures in bold have been described in the paragraph above.

## Supplementary Materials

NMR spectra for compounds **1**–**4** as well as computational data for compound **1** are available online at http://www.mdpi.com/1660-3397/16/2/61/s1 in Figures S1–S31 and Tables S1 and S2.
