# Supplementary material for: Chlorinated Azaphilone Pigments with Antimicrobial and Cytotoxic Activities Isolated from the Deep Sea Derived Fungus Chaetomium sp. NA-S01-R1
Source: Mar Drugs. 2018 Feb 13;16(2):61. doi: 10.3390/md16020061 (PMC5852489; doi:10.3390/md16020061)
Supplement: Supplementary file 1 [file marinedrugs-16-00061-s001.pdf]

Supplementary Files

Chlorinated azaphilone pigments with antimicrobial and cytotoxic activities isolated from the deep sea derived Fungus *Chaetomium* sp. NA-S01-R1

Weiyi Wang [1,*], Yanyan Liao [2], Ruixuan Chen [1], Yanping Hou [1], Wenqian Ke [1], Beibei Zhang [1], Maolin Gao [1], Zongze Shao [1], Jianming Chen [3,*] and Fang Li [1,*]

Content

Figure S1. ^{1}H NMR spectrum of compound 1
Figure S2. ^{13}C NMR spectrum of compound 1
Figure S3. HSQC spectrum of compound 1
Figure S4. HMBC spectrum of compound 1
Figure S5. HRESIMS spectrum of compound 1
Figure S6. ^{13}C/DEPT spectrum of compound 1
Figure S7. ^{1}H-^{1}H COSY spectrum of compound 1
Figure S8. Selected NOESY spectrum of compound 1
Figure S9. Selected NOESY spectrum of compound 1
Figure S10. ^{1}H NMR spectrum of compound 2
Figure S11. ^{13}C NMR spectrum of compound 2
Figure S12. HSQC spectrum of compound 2
Figure S13. HMBC spectrum of compound 2
Figure S14. HRESIMS spectrum of compound 2
Figure S15. ^{13}C/DEPT spectrum of compound 2
Figure S16. ^{1}H-^{1}H COSY spectrum of compound 2
Figure S17. NOESY spectrum of compound 2
Figure S18. ^{1}H NMR spectrum of compound 3
Figure S19. ^{13}C NMR spectrum of compound 3
Figure S20. HSQC spectrum of compound 3
Figure S21. HMBC spectrum of compound 3
Figure S22. HRESIMS spectrum of compound 3
Figure S23. ^{13}C/DEPT spectrum of compound 3
Figure S24. ^{1}H-^{1}H COSY spectrum of compound 3
Figure S25. ^{1}H NMR spectrum of compound 4
Figure S26. ^{13}C NMR spectrum of compound 4
Figure S27. HSQC spectrum of compound 4
Figure S28. HMBC spectrum of compound 4
Figure S29. HRESIMS spectrum of compound 4
Figure S30. ^{13}C/DEPT spectrum of compound 4
Figure S31. ^{1}H-^{1}H COSY spectrum of compound 4
Table S1; Table S2; Text S1

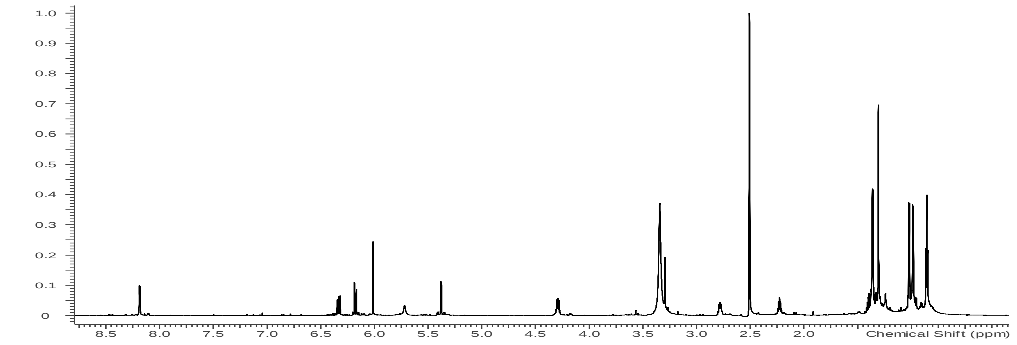

Figure S1. ^{1}H NMR spectrum of compound 1

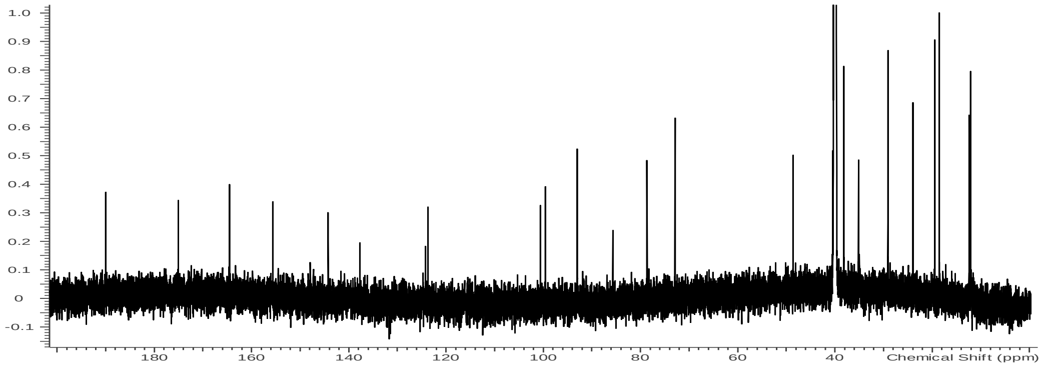

Figure S2. ^{13}C NMR spectrum of compound 1

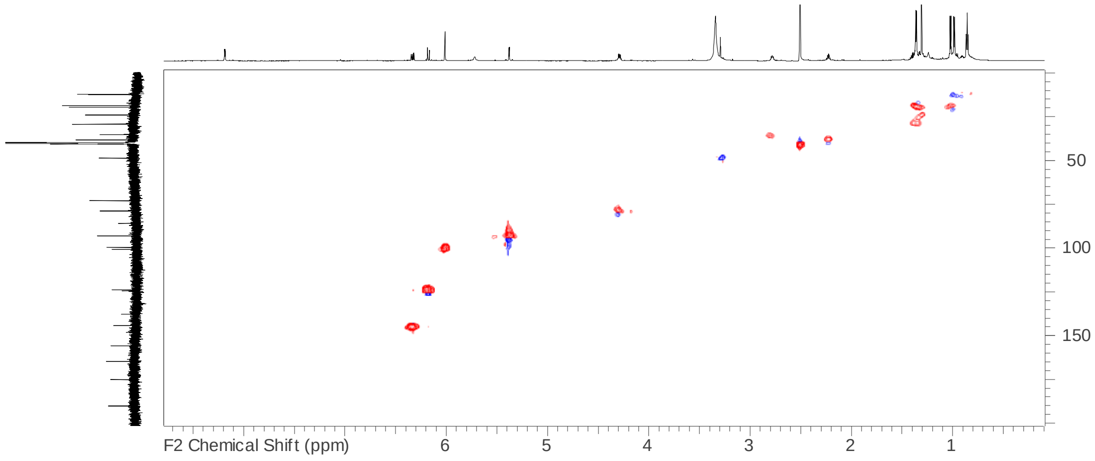

Figure S3. HSQC spectrum of compound 1

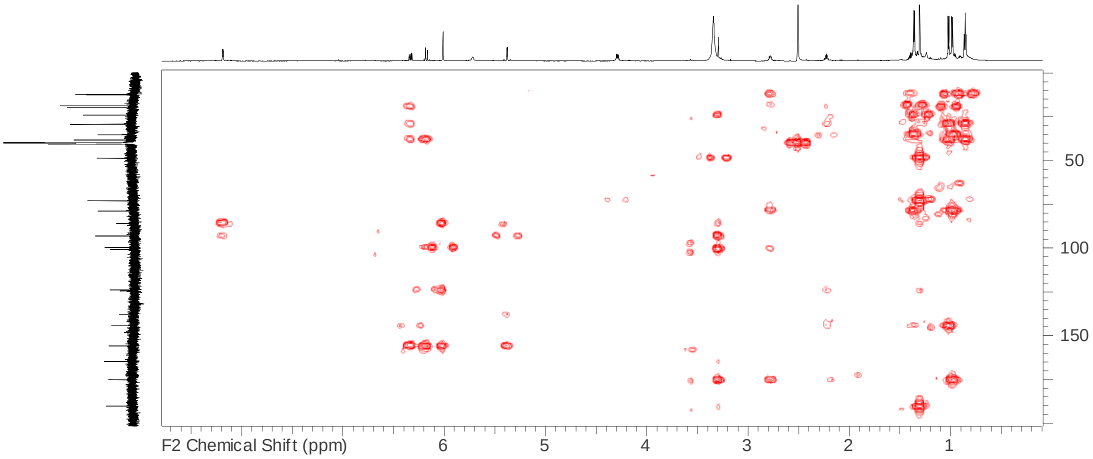

Figure S4. HMBC spectrum of compound 1

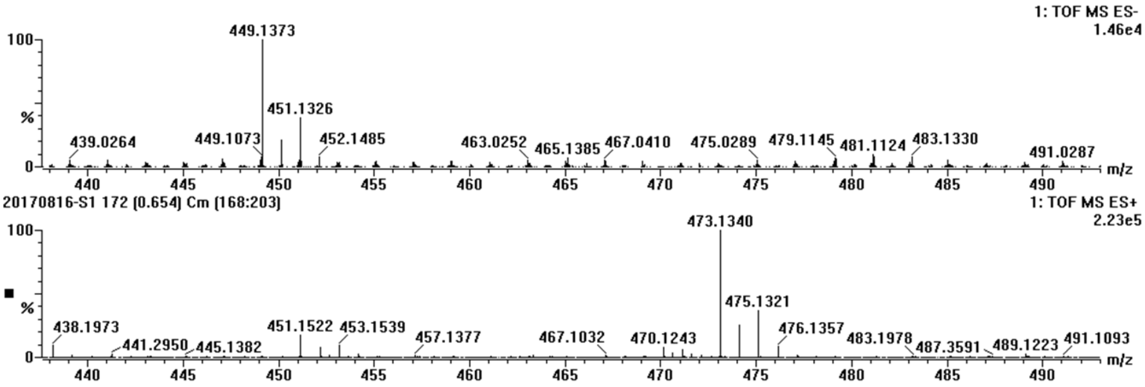

Figure S5. HRESIMS spectrum of compound 1

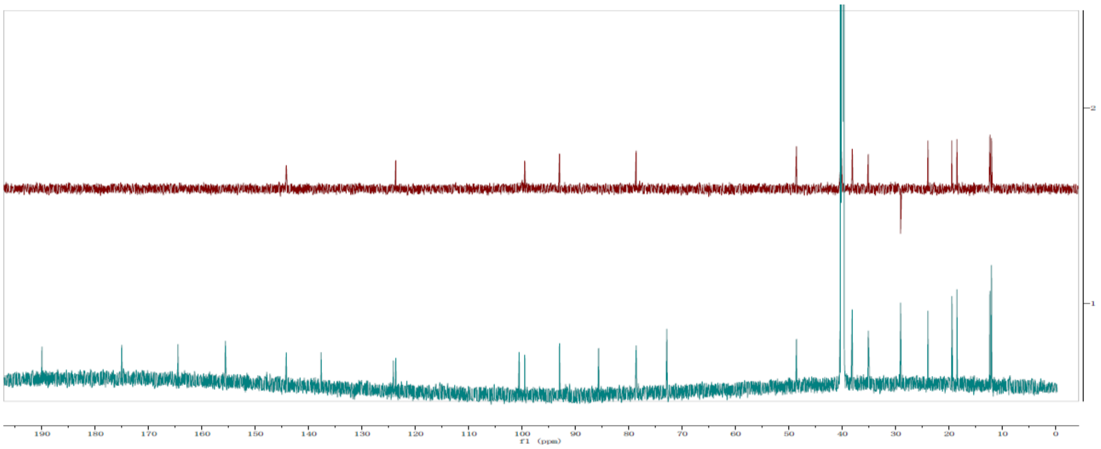

Figure S6. ^{13}C/DEPT spectrum of compound 1

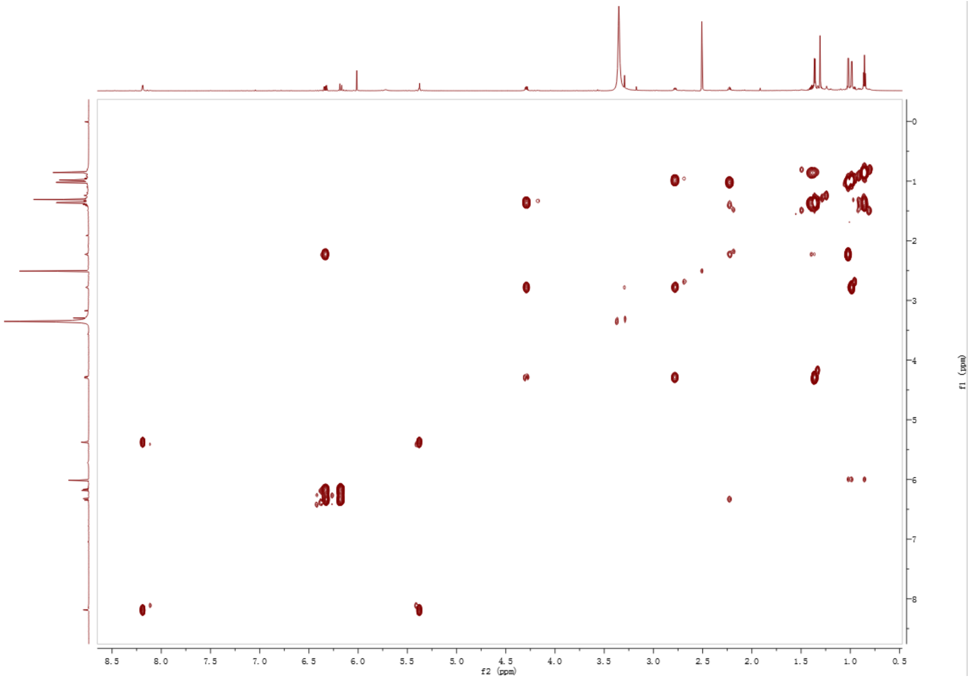

Figure S7. ^{1}H-^{1}H COSY spectrum of compound 1

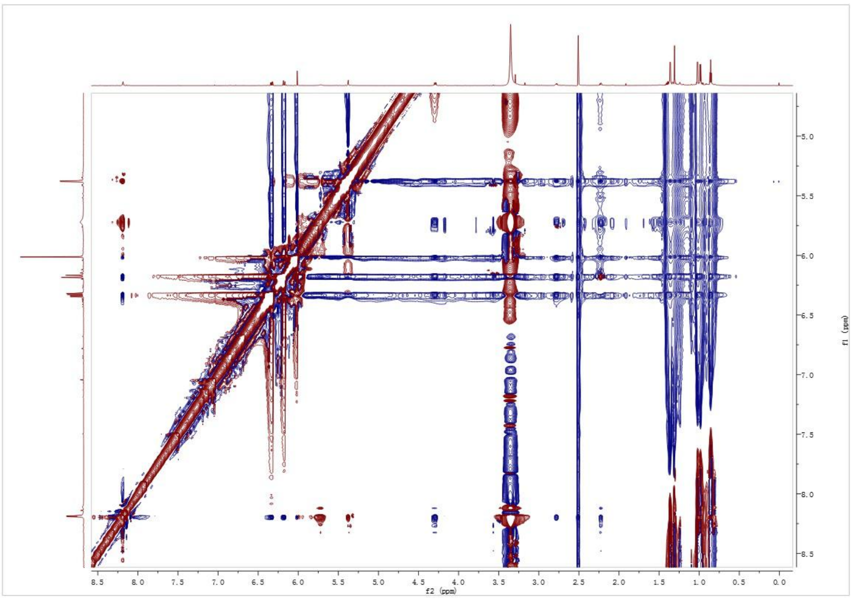

Figure S8. Selected NOESY spectrum of compound 1

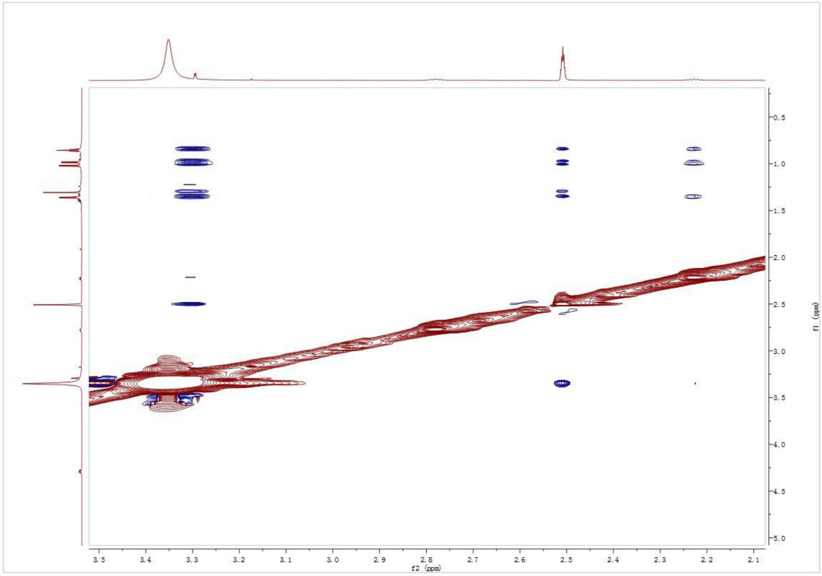

Figure S9. Selected NOESY spectrum of compound 1

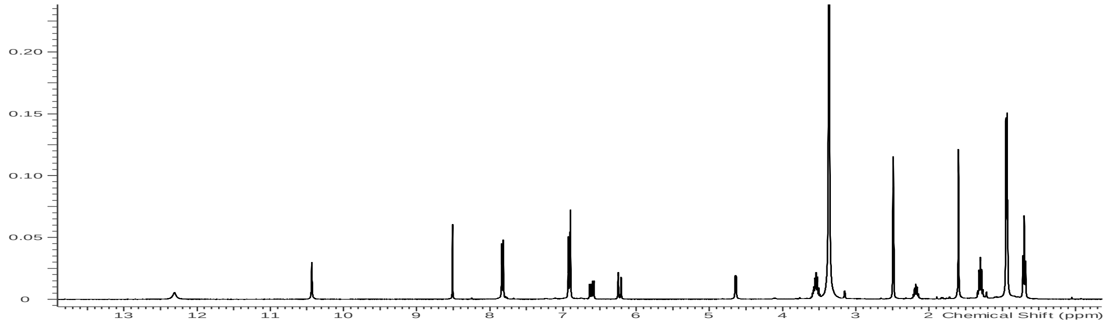

Figure S10. ^{1}H NMR spectrum of compound 2

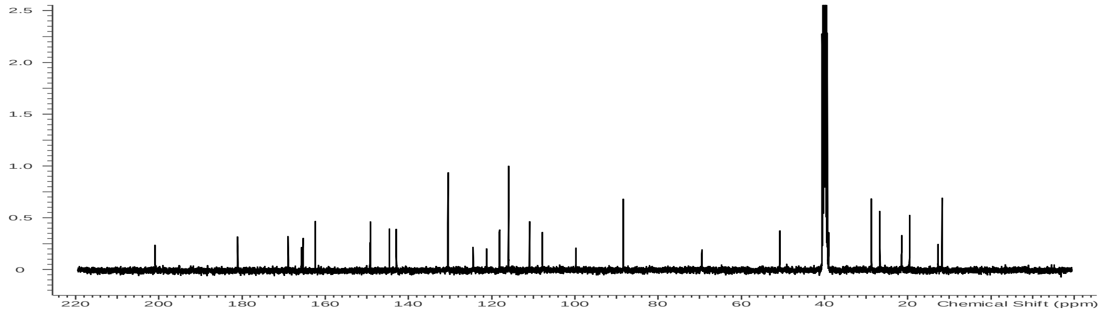

Figure S11. ^{13}C NMR spectrum of compound 2

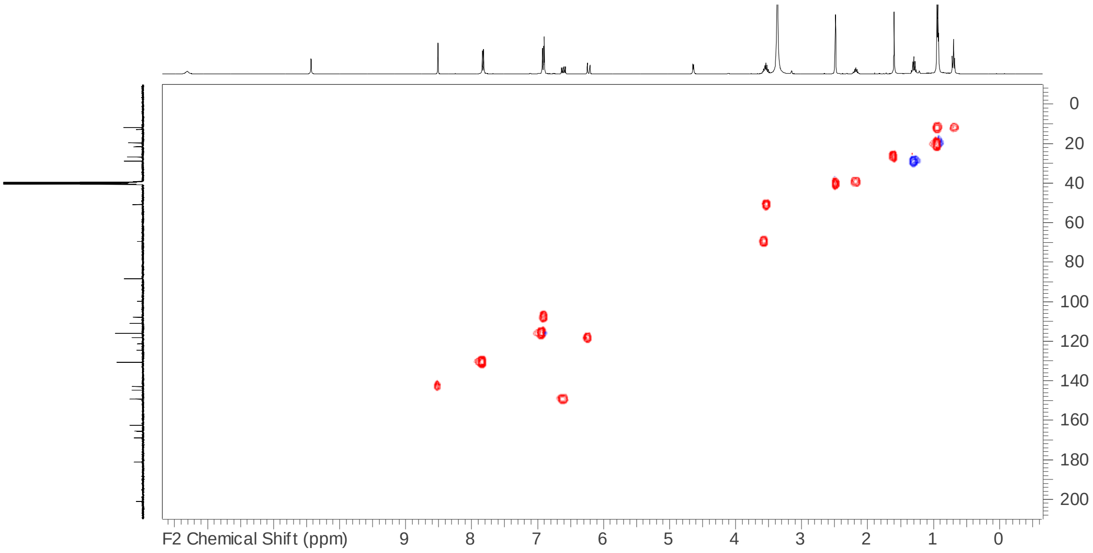

Figure S12. HSQC spectrum of compound 2

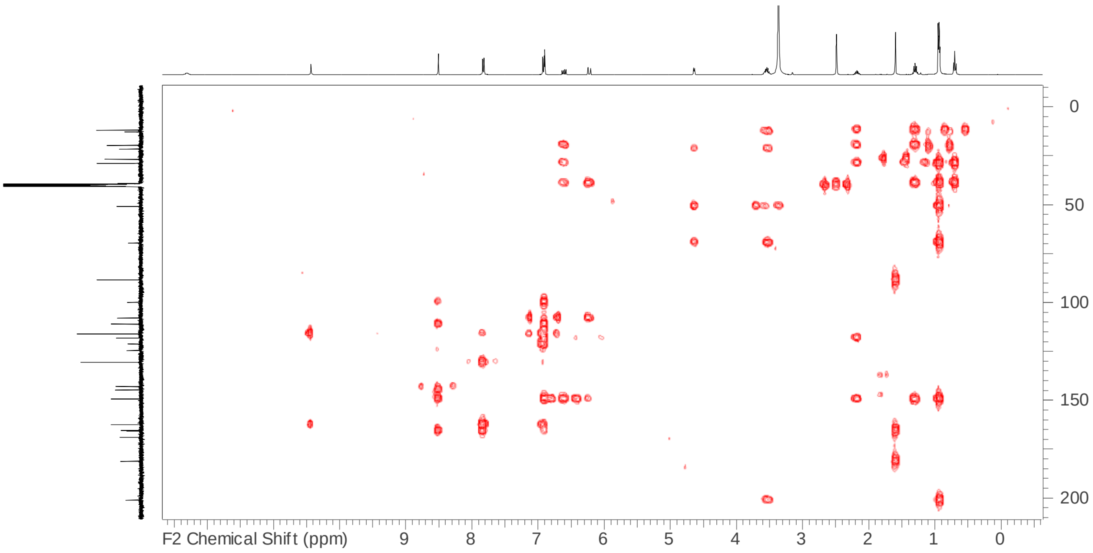

Figure S13. HMBC spectrum of compound 2

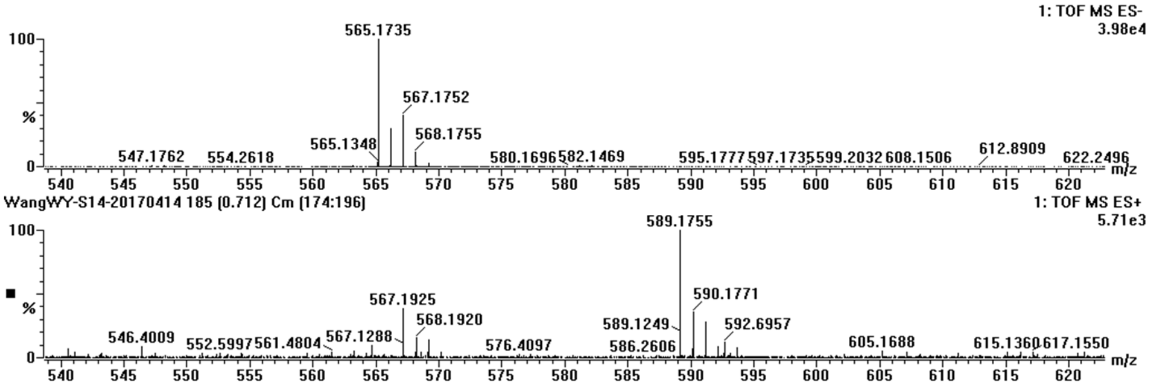

Figure S14. HRESIMS spectrum of compound 2

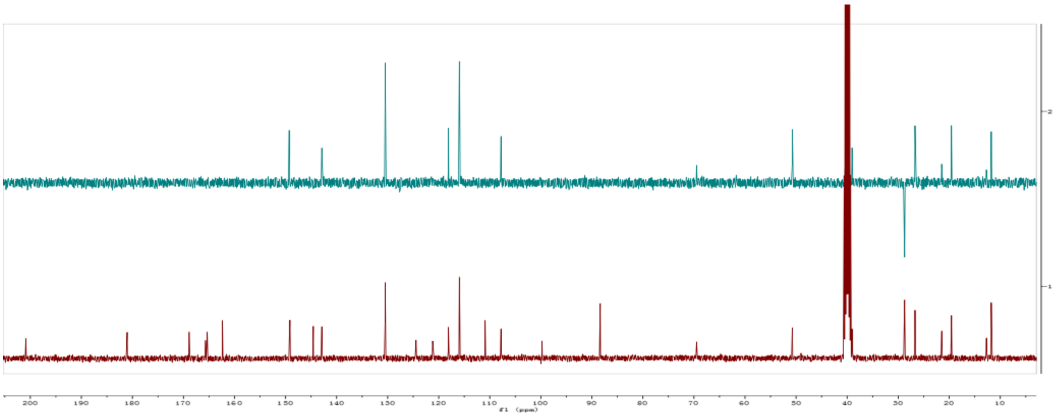

Figure S15. ^{13}C/DEPT spectrum of compound 2

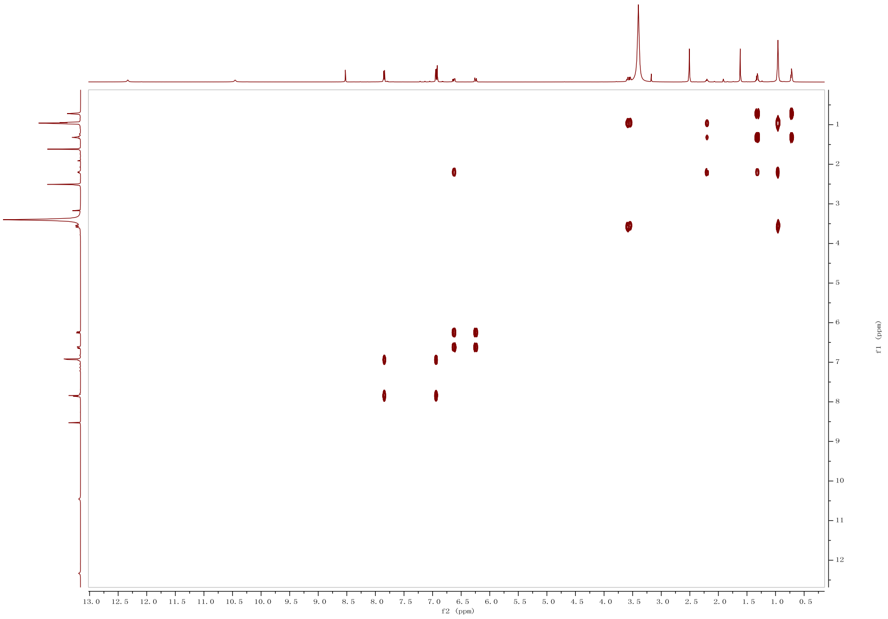

Figure S16. ^{1}H-^{1}H COSY spectrum of compound 2

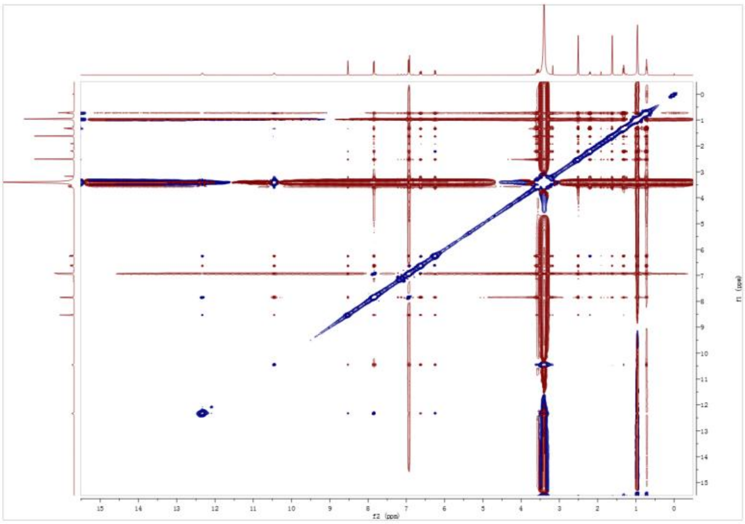

Figure S17. NOESY spectrum of compound 2

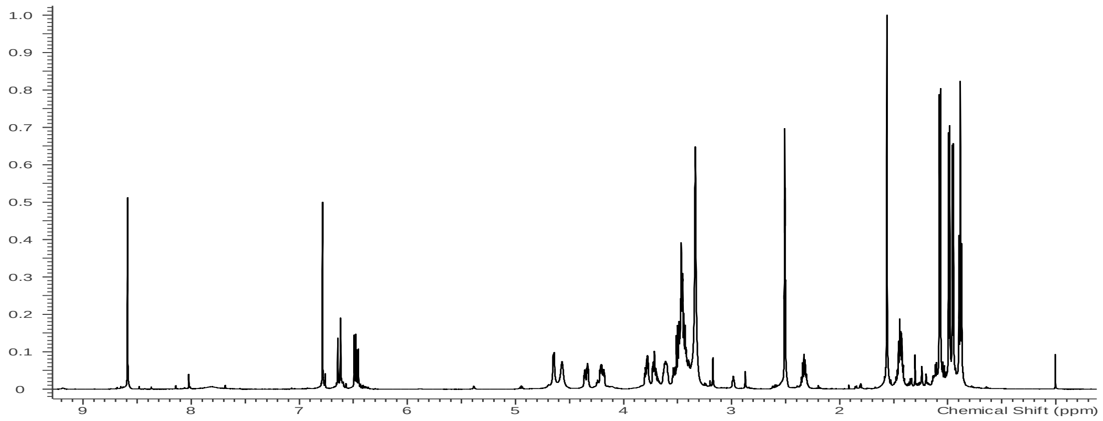

Figure S18. ^{1}H NMR spectrum of compound 3

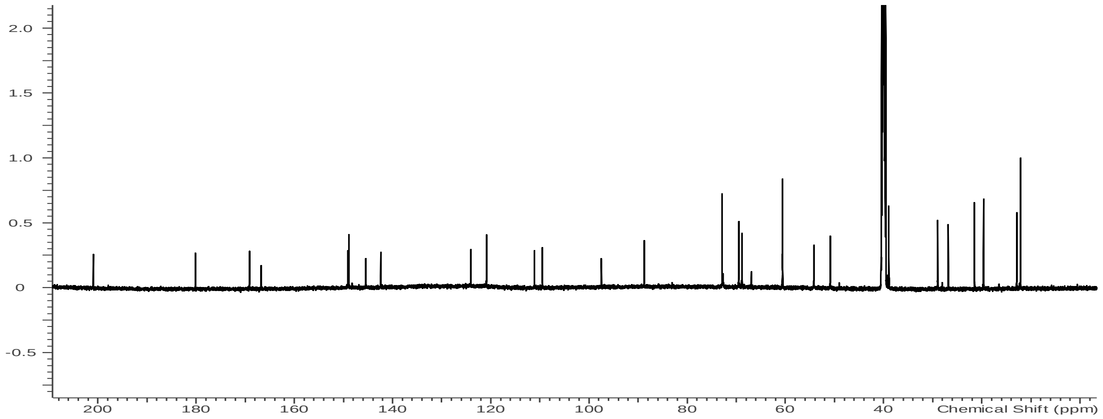

Figure S19. ^{13}C NMR spectrum of compound 3

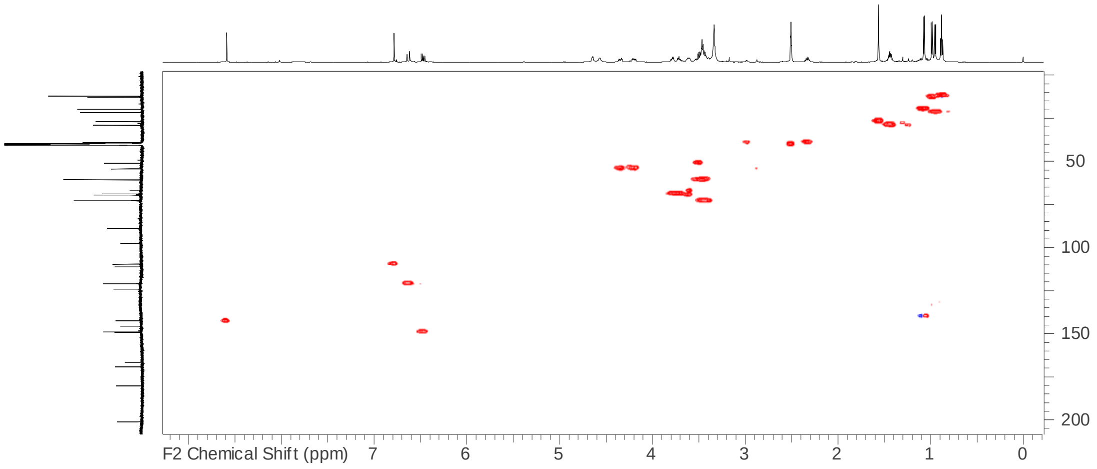

Figure S20. HSQC spectrum of compound 3

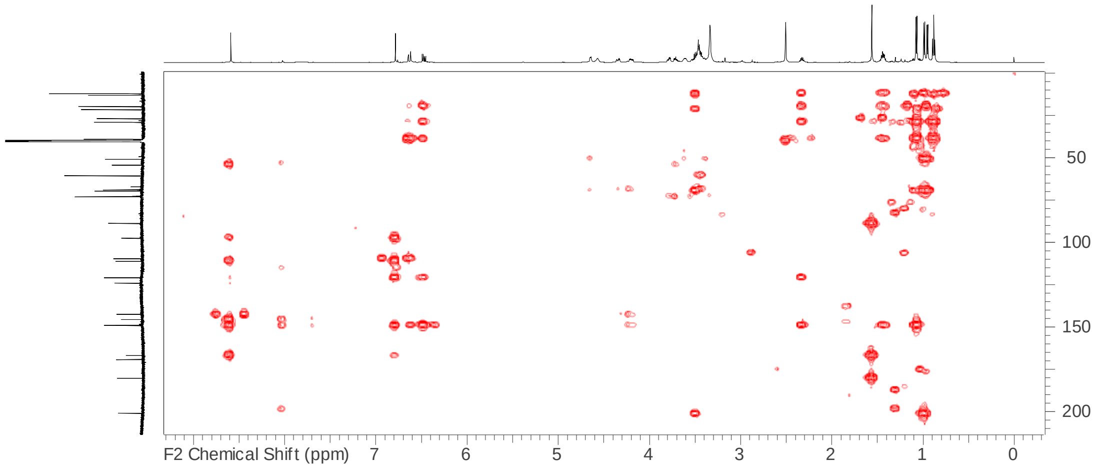

Figure S21. HMBC spectrum of compound 3

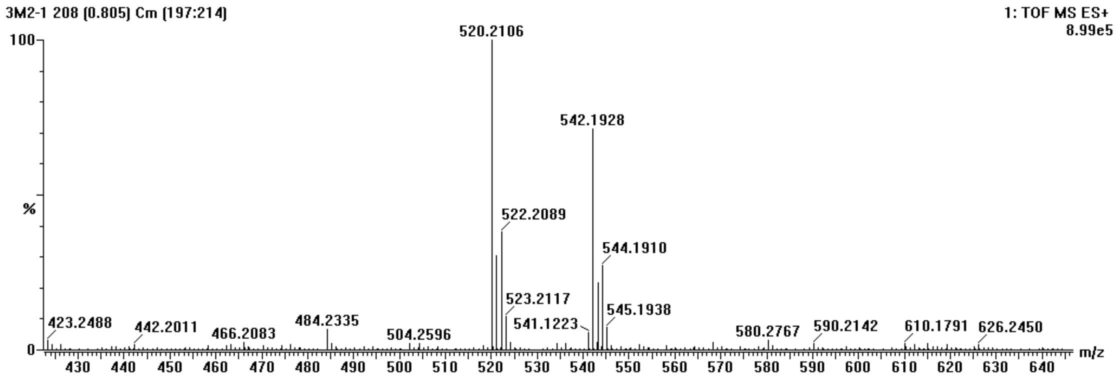

Figure S22. HRESIMS spectrum of compound 3

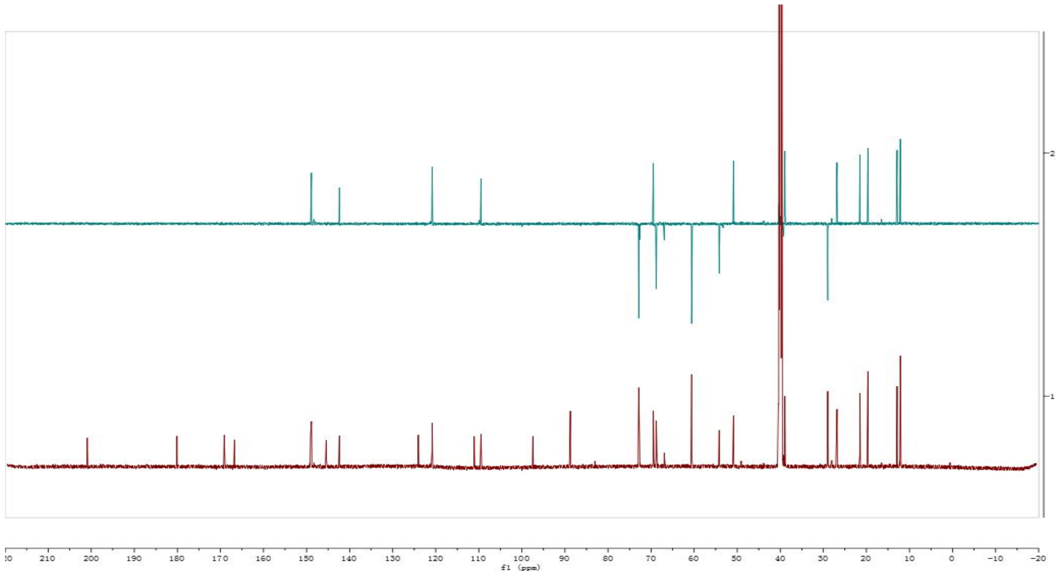

Figure S23. ^{13}C/DEPT spectrum of compound 3

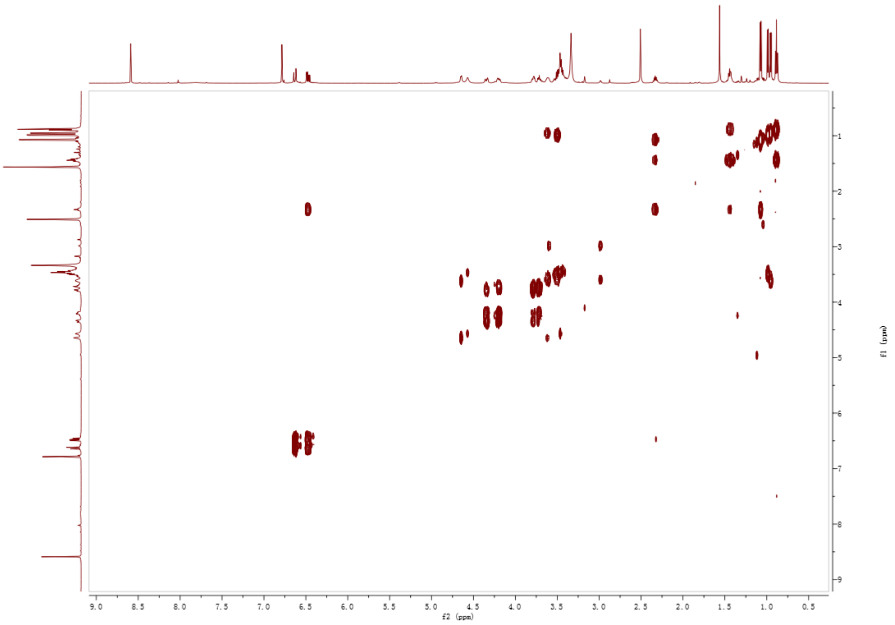

Figure S24. ^{1}H-^{1}H COSY spectrum of compound 3

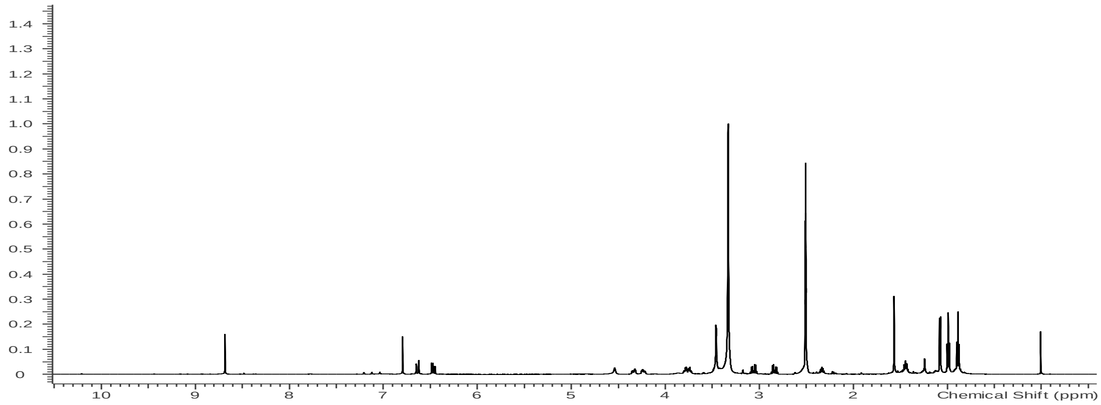

Figure S25. ^{1}H NMR spectrum of compound 4

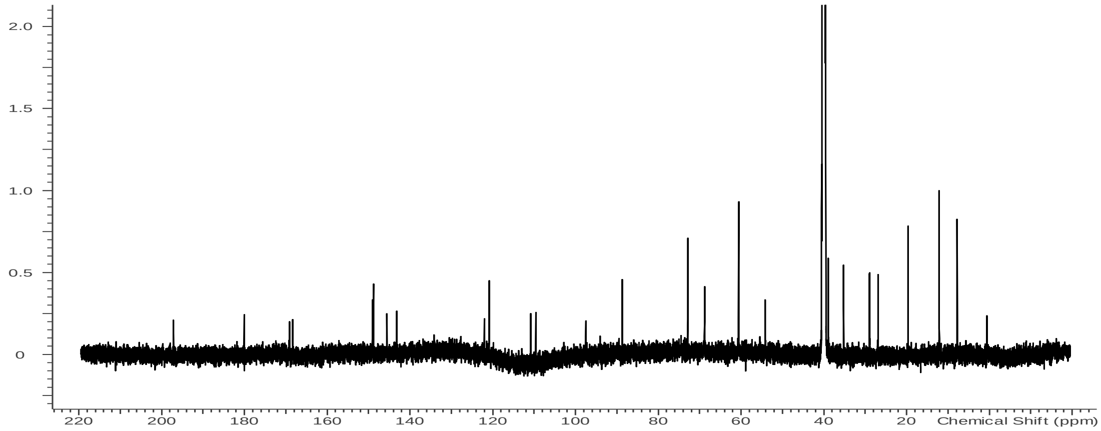

Figure S26. ^{13}C NMR spectrum of compound 4

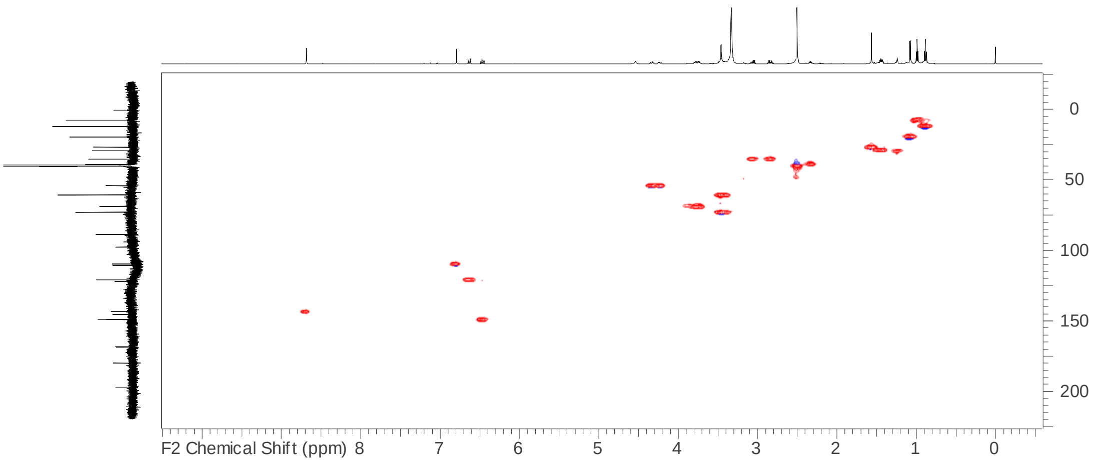

Figure S27. HSQC spectrum of compound 4

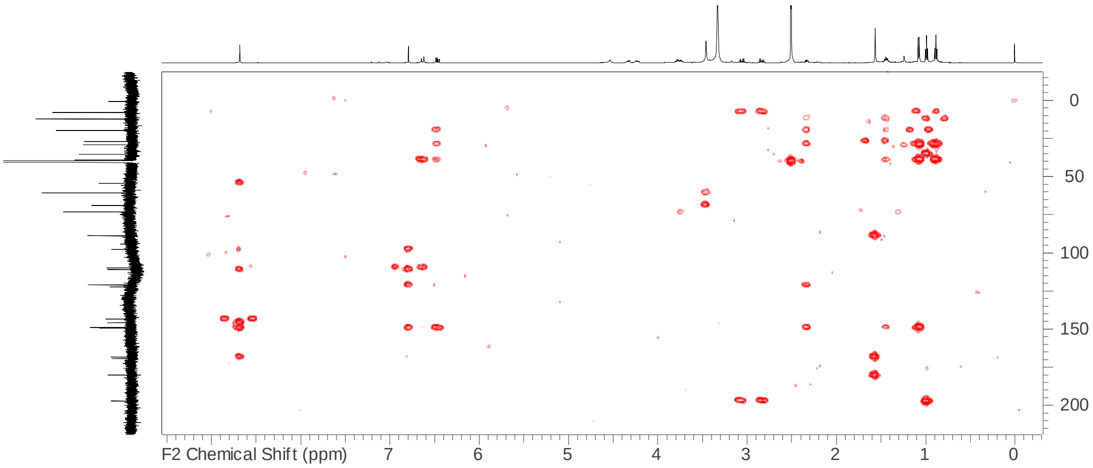

Figure S28. HMBC spectrum of compound 4

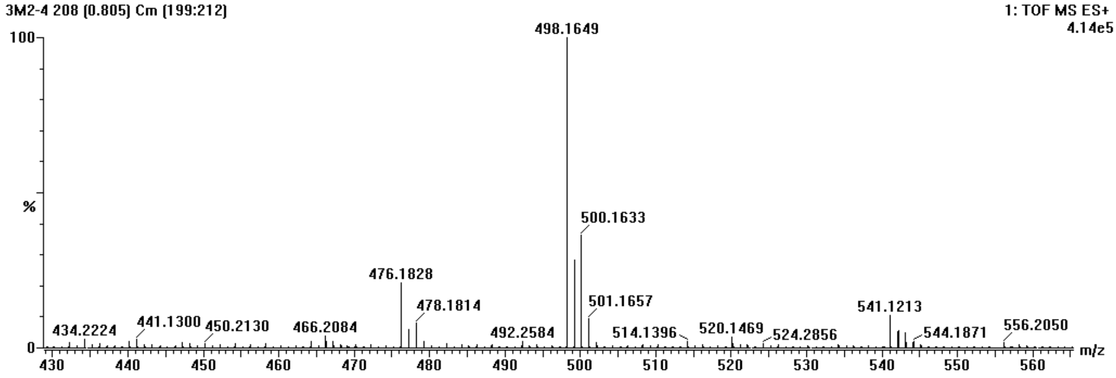

Figure S29. HRESIMS spectrum of compound 4

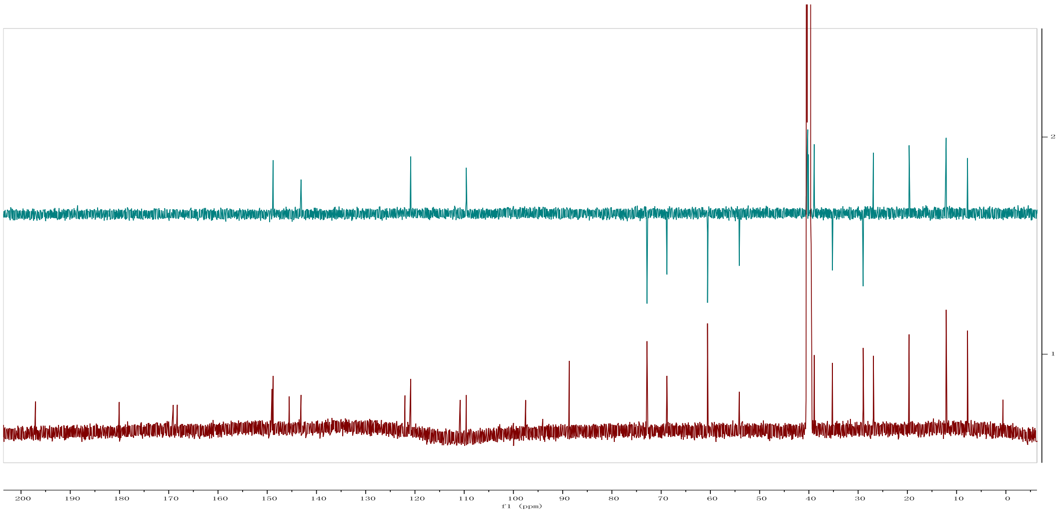

Figure S30. ^{13}C/DEPT spectrum of compound 4

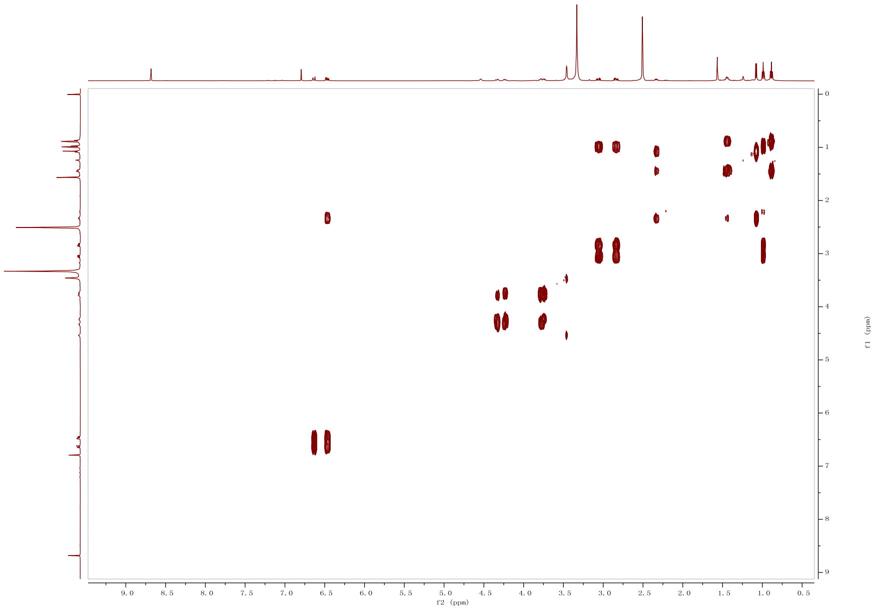

Figure S31. ^{1}H-^{1}H COSY spectrum of compound 4

Table S1. Energies of the dominative conformers at MMFF94 force field of compound 1

Configuration	Conformer	Energy (kcal/mol)
(1*R*, 7*R*, 8*S*, 8a*R*, 11*S*, 4′*R*, 5′*S*)-**1**	1	131.80
	2	133.62
	3	133.64
	4	133.71
(1*S*, 7*S*, 8*R*, 8a*S*, 11*S*, 4′*S*, 5′*R*)-**1**	1	134.38
	2	135.20
	3	135.64
	4	135.94
	5	135.95
	6	136.04

		7	136.16

Table S2. Energies of the conformers at B3LYP/6-311G** of compound 1 in methanol.

Configuration	Conformer	Structure	E (Hartree)	E (kcal/mol)	Population (%)
(1*R*, 7*R*, 8*S*, 8a*R*, 11*S*, 4′*R*, 5′*S*)-**1**	3	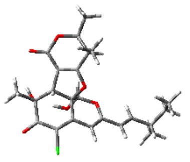	-1879.82988243	-1179611.05	100.00
(1*S*, 7*S*, 8*R*, 8a*S*, 11*S*, 4′*S*, 5′*R*)-**1**	1	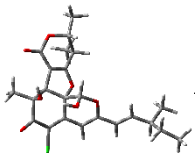	-1879.82899701	-1179610.50	26.34
(1*S*, 7*S*, 8*R*, 8a*S*, 11*S*, 4′*S*, 5′*R*)-**1**	2	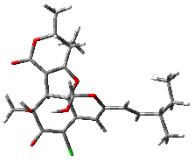	-1879.82633158	-1179608.82	1.57
(1*S*, 7*S*, 8*R*, 8a*S*, 11*S*, 4′*S*, 5′*R*)-**1**	4	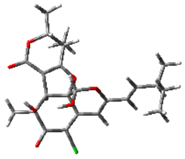	-1879.82994775	-1179611.09	72.10

Text S1: ITS1-5.8S-ITS2 rDNA sequence (MG786198) of strain *Chaetomium* sp. NA-S01-R1

LOCUS Seq 568 bp DNA linear PLN 11-JAN-2018
DEFINITION 1 Chaetomium sp. NA-S01-R1 18S ribosomal RNA gene, partial
sequence; internal transcribed spacer 1, 5.8S ribosomal RNA gene,
and internal transcribed spacer 2, complete sequence; and 28S
ribosomal RNA gene, partial sequence.
ACCESSION Seq
VERSION
KEYWORDS .
SOURCE Chaetomium sp.
ORGANISM Chaetomium sp.
Eukaryota; Fungi; Dikarya; Ascomycota; Pezizomycotina;
Sordariomycetes; Sordariomycetidae; Sordariales; Chaetomiaceae;
Chaetomium.
REFERENCE 1 (bases 1 to 568)
AUTHORS Wang, W.
TITLE Chlorinated azaphilone pigments with antimicrobial and cytotoxic

```
            activities isolated from the deep sea derived Fungus Chaetomium sp.
            NA-S01-R1
  JOURNAL   Unpublished
REFERENCE   2  (bases 1 to 568)
  AUTHORS   Wang, W.
  TITLE     Direct Submission
  JOURNAL   Submitted (11-JAN-2018) Key Laboratory of Marine Biogenetic
            Resources, Third Institute of Oceanography, State Oceanic
            Administration, 178 Daxue Road, Xiamen, Fujian 361005, China
COMMENT     Bankit Comment: ALT EMAIL:wywang_cas@163.com.
            Bankit Comment: TOTAL # OF SEQS:1.

            ##Assembly-Data-START##
            Sequencing Technology :: Sanger dideoxy sequencing
            ##Assembly-Data-END##
FEATURES             Location/Qualifiers
     source          1..568
                     /organism="Chaetomium sp."
                     /mol_type="genomic DNA"
                     /isolate="NA-S01-R1"
                     /isolation_source="seawater"
                     /bio_material="Chaetomium sp."
                     /db_xref="taxon:1769349"
                     /collection_date="July 2017"
                     /collected_by="Yanping Hou"
                     /identified_by="Weiyi Wang"
     misc_RNA        1..568
                     /note="contains 18S ribosomal RNA gene, partial sequence;
                     internal transcribed spacer 1, 5.8S ribosomal RNA gene,
                     and internal transcribed spacer 2, complete sequence; and
                     28S ribosomal RNA gene, partial sequence"
BASE COUNT      125 a    150 c    163 g    130 t
ORIGIN
        1 ctggcggtct ctacttttcc gaggtcacct tgggttaaaa ggtggtttaa cggccggaac
       61 ccgcggcgcg accagagcga gatgtatgct actacgctcg gtgcgacagc gagcccgcca
      121 ctgcttttca gggcctgcgg cagccgcagg tccccaacac aagcccgggg gcttgatggt
      181 tgaaatgacg ctcgaatagg catgcccgcc agaatgctgg cgggcgcaat gtgcgttcaa
      241 agattcgatg attcactgaa ttaagcaatt cacattactt atcgcatttc gctgcgttct
      301 tcatcgatgc cagaaccaag agatccgttg ttgaaagttt tgacttattc agtacagaag
      361 actcagagag gccagaaatt atcaagagtt tggtgaccgc cggcgggcgc ccgcggtggg
      421 gcccaggggc gcccggaggg taaaccccgg ggccgcccgc cgaagctacg gtataggtaa
      481 cgttcacaat ggtttaggga gttttgcaac tctgtaatga tccctccgct ggttcaccaa
      541 cggagacctt gttacgattt tttacttc
//
```
